# A simplified relationship between the modified O-lattice and the rotation matrix for generating the coincidence site lattice of an arbitrary Bravais lattice system

**DOI:** 10.1107/S2053273322000171

**Published:** 2022-02-18

**Authors:** Hongwei Liu

**Affiliations:** a The Australian Centre for Microscopy and Microanalysis, The University of Sydney, City Road, Sydney, NSW 2077, Australia

**Keywords:** grain boundaries, phase transitions, coincidence site lattice, CSL, near coincidence site lattice, NCSL, rotation matrix, O-lattice

## Abstract

The coincidence site lattice (CSL) is important for characterizing the structure and energy state of grain boundaries in polycrystalline materials. A simplified relationship between the modified O-lattice and the corresponding rotation matrix is proposed to establish a general formula for the CSL and the near coincidence site lattice (NCSL) in Bravais lattice systems. The general formula paves the way to computer simulation and crystallographic analysis of grain boundaries.

## Abstract 2

A simplified relationship between the modified O-lattice and the rotation matrix of any Bravais lattice was established for the generation of a coincidence site lattice wherever it exists.

## 1. Introduction

The coincidence site lattice (CSL) concept was derived by Ranganathan on the hypothesis that there are certain common sites located on a single lattice of larger cell dimensions compared with two adjacent identical crystal lattices related by a special rotation operation (Ranganathan, 1966 ▸). Tables of CSLs in cubic lattices have been reported independently by Warrington & Bufalini (1971 ▸), Bleris & Delavignette (1981 ▸) and Grimmer (1984 ▸). The rotation operation and multiplicity of a CSL are determined by the rotation axis [*uvw*] and the rotation angle θ (Ranganathan, 1966 ▸). The CSL formulation was then characterized mathematically for the general case by Santoro & Mighell (1973 ▸). The CSL concept was also extended into reciprocal space to propose displacement shift complete (DSC) lattices (Grimmer *et al.*, 1974 ▸). More complicated systems, such as face- and body-centred cubic crystals, have been discussed in terms of near coincidence site lattices (NCSLs) and DSC lattices for cubic systems based on the O-lattice concept (Grimmer *et al.*, 1974 ▸; Bonnet *et al.*, 1981 ▸; Balluffi *et al.*, 1982 ▸) and in terms of NCSLs for hexagonal systems (Bleris *et al.*, 1982 ▸).

Ranganathan’s formula gives a simple and fast criterion for the determination of the existence of a CSL for a given axis or a given multiplicity Σ of a simple cubic crystal. Grimmer’s method has the advantage of being a more systematic method for all cubic systems. Bleris’s new formulation produced a systematic generation of CSLs, which was successfully extended to the hexagonal system. However, the tables of CSLs reported in literature were limited to the cubic system (Warrington & Bufalini, 1971 ▸) and the hexagonal system (Bonnet *et al.*, 1981 ▸). The general treatment for an arbitrary system provided by Santoro showed no details for CSLs for different Bravais systems (Santoro & Mighell, 1973 ▸) owing to the difficulty of finding perfect superimposed lattice sites from two correlated non-cubic lattices.

In the last few decades, some new formulations have been proposed for CSLs of cubic structures, such as the geometric method for two-dimensional (2D) CSLs (Shamsuzzoha & Rahman, 2012 ▸), the 3D CSL method based on Grimmer’s reciprocity theorem and the reduction algorithm (Lord, 2006 ▸). However, it is accepted that there are some major difficulties in extending the CSL concept from cubic to non-cubic crystals (Fortes, 1977 ▸), although the new models mentioned above are still valid for pure cubic, hexagonal and even some monoclinic systems. For instance, NCSLs for hexagonal structures were investigated by Bonnet *et al.* (1981 ▸) for a range of metals for the [0001], 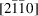 and 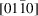 zone axes and NCSLs for monoclinic structures were initially considered by Gertsman *et al.* (1996 ▸).

A general and easy-to-use model of NCSLs and CSLs is essential for computer simulations. A few applications are the simulation of hetero-epitaxial interface structures (Sayle *et al.*, 1993 ▸), the three-dimensional interface between α-Ti and β-Ti of titanium alloys (Miyano *et al.*, 2000 ▸), and orientation relationships and interface structure of dual-phase alloys (Miyano & Ameyama, 2000 ▸).

As most of the reported CSL and NCSL models have been generated from simple cubic lattices, it was necessary to build a model to meet the requirements of more complicated, mixed structures, such as base-centred, body-centred and face-centred lattices. Although Grimmer proposed a method to allow calculating the CSL and NCSL for two arbitrary lattices (Grimmer, 1989 ▸), it started from the reciprocal lattice. This method was based on phase transformations, so that a computer-aided automatic method could deduce possible CSLs and NCSLs of a phase transformation system.

In early work on grain boundary analysis by Karakostas *et al.* (1979 ▸), a modified O-lattice method was proposed to generate a CSL and DSC lattice for an arbitrary Bravais lattice. This is a simple and relatively easy to use model for CSLs. A crystal I in a coordinate system (denoted as W_1_) is transformed to a crystal II by a rotation matrix ***R*** = [*p*_1_ *p*_2_ *p*_3_]/θ, where *p*_1_, *p*_2_ and *p*_3_ are the direction cosines of the reference coordination system (denoted as V_1_), and θ is the rotation angle of the rotation axis [*uvw*]. A reference system has its *Z* axis parallel to the rotation axis and the other two axes lying in the reference plane. Then the modified O-lattice vectors **x** ^(O)^ in a cubic lattice are expressed in W_1_ as

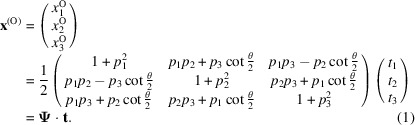




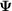
 denotes the conversion matrix of the O-lattice, and **t** are the modified translation vectors.

This is a modified O-lattice because the translation vectors **t** in equation (1) are not lattice translation vectors **b** ^L^ for crystal I. It is a new lattice defined by two vectors in the reference plane and the third vector parallel to the rotation axis with length equal to the modulus of the rotation axis, *i.e.*, the distance between two adjacent lattice points lying on this axis. That is to say, the vector **b** ^L^ is use for generating the real O-lattice while **t** is used for modified O-lattice calculations. For example, the translation vector of crystal I and the vector for the modified O-lattice rotation around [111] are

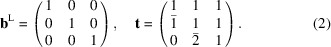

This can be extended to a non-cubic lattice by applying the transformation matrix ***S*** from the crystal system being investigated to the orthonormal reference system,

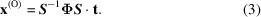

However, equation (1) is complicated and not easy to use, especially in the case of low-symmetry lattices, say for hexagonal, orthorhombic and monoclinic structures. It would be very helpful to find a way to simplify this formulation.

In this work it is found that there is a direct link between the modified O-lattice and the rotation matrix ***R***. A simplified O-lattice formula for CSLs is developed accordingly. In the following, the proposed formula is presented first, followed by details of how it was developed and applications to real cases of CSLs of high-symmetry lattices and NCSLs of medium- and low-symmetry lattices.

## 2. Simplification of the modified O-lattice

For a rotation matrix ***R*** = [*p*_1_, *p*_2_ *p*_3_]/θ, let *a* and *b* be the values of cos θ and sin θ, where θ is the rotation axis and ***I*** represents the unit matrix, *i.e.*,

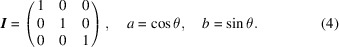

Then the conversion matrix 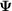 for generating the modified O-lattice matrix shown in equation (1) can be solved as

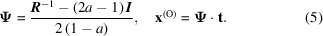

For non-cubic lattices, this becomes

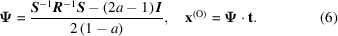

The simplified O-lattice formulae shown in equations (5) and (6) suggest that the modified O-lattice is the product of a rotation matrix inversion with a translation matrix, which is then rescaled. Thus, the simplified O-lattice formula illustrates a clear geometric meaning and is easy to calculate by hand or by computer.

## 3. Theoretical development of the simplified O-lattice of a CSL for a cubic lattice

For a given cubic crystal I, the three basis axes are parallel to a reference Cartesian coordinate system W_1_ and are denoted as **X**, **Y** and **Z**. The CSL generated by a lattice rotation around an axis [*uvw*] with a rotation angle θ can be obtained by the O-lattice method. Note that the 3D O-lattice of a pure rotation matrix has no roots, as the determinant of the total strain is zero:

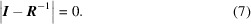

This property makes it inconvenient to produce an O-lattice from a pure rotation matrix by following the classical O-lattice concept. To find the solution, it is necessary to calculate a two-dimensional case, which is deduced from the 3D case.

### 3.1. The modified O-lattice generated by the O-lattice method

Consider a rotation operation around an arbitrary axis **u** as [*u* *v* *w*] with arbitrary angle θ. The rotation matrix ***R*** has the general form

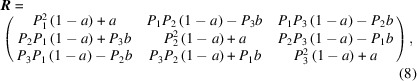

where the parameters *a* and *b* were defined in Section 2. The rotation axis [*u* *v* *w*] is unitized as [*p*_1_, *p*_2_, *p*_3_] where

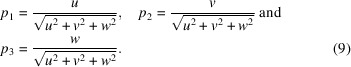

Consider a new coordinate system V_1_, where 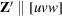 , 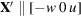 and 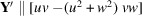 . The rotation matrix that converts the old system W_1_ to the new system V_1_ is

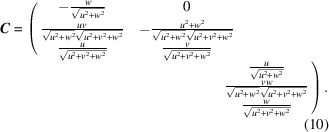

In this new system V_1_, the original 3D rotation can be simplified to a 2D rotation around the **Z**′ axis with the same rotation angle θ. The rotation matrix ***Q*** for [001]/θ in two dimensions is

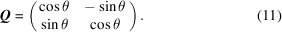

The 2D O-lattice solution gives

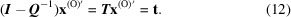


**t** in the V_1_ system is identical to **b** ^L^,

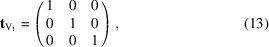

and the O-lattice in 2D space is

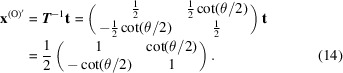

The multiplicity of the O-lattice for the primitive cubic unit cell is

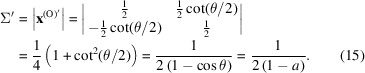

Since a CSL is a superlattice of the O-lattice, the multiplicity Σ of a CSL must be an integer (*n*) times that of the O-lattice:

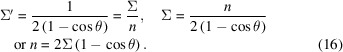

By applying a reverse rotation ***C*** ^−1^,

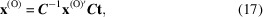




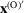
 will be converted into the O-lattice **x** ^(O)^ in three dimensions again:

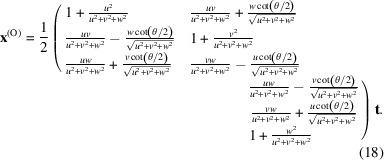


**t** in the W_1_ system is

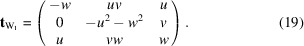

Replacing the directional cosine values shown in equation (9), equation (18) can be rewritten as

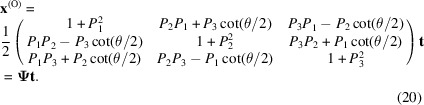

This is the same as equation (1). To validate the equation, consider the 2D case. When *u* = *v* = 0 and *w* = 1, equation (20) gives

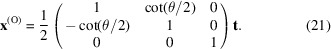

This is the same as equation (14).

### 3.2. The modified O-lattice generated by re-forming the rotation matrix

We can, however, re-form the rotation matrix and obtain the same modified O-lattice with some interesting findings. The inversion of the rotation matrix ***R*** can be expressed as

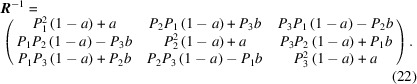

It can be further dissociated into the following form:

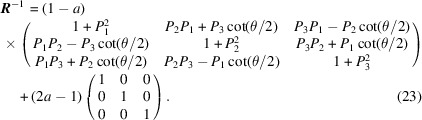

When revisiting equation (20), it is interesting to find that the matrix for generating the O-lattice from a pure rotation has a very similar form to that of the rotation matrix. Substituting the known items in equation (20) into equation (23), it then reads

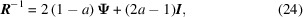

from which the conversion matrix 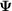 for generating the modified O-lattice can be solved:

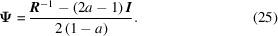

**Figure 1:**
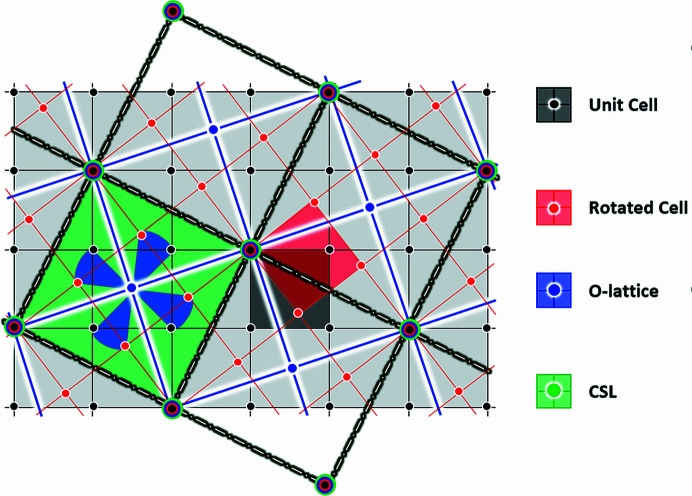
The 2D geometric relationships between a unit cell, the CSL rotation cell, the O-lattice and the CSL in a cubic crystal, showing that the CSL sites are the superimposed sites of the unit cell, the rotation cell and the O-lattice.

This equation reveals a simplified relationship between the modified O-lattice for a CSL and the rotation matrix. That means a CSL can now be mathematically re-formed by using the rotation matrix directly without encountering the issue of zero determinant shown in equation (7). It can be graphically illustrated as in Fig. 1 ▸ for example, which shows the geometric relationships between a unit cell, its rotated cell, the O-lattice and the CSL (Σ5 = [100]/36.87°) of a cubic crystal.

### 3.3. Extension into general Bravais lattice systems

Following the mathematical method used in this work, it is not difficult to extend the above inferences to all the seven lattice systems. It is known that the Niggli reduced cell contains only one lattice site occupied by an atom or a group of atoms. The six lattice parameters of an arbitrary Niggli reduced cell are *a*_0_, *b*_0_, *c*_0_, α, β and γ. The *a* axis is parallel to the *X* axis and the plane determined by the cross product of the *a* and *b* axes is parallel to the **XOY** plane of the reference coordinate system. For an arbitrary triclinic crystal, its transformation matrix ***S*** is expressed as

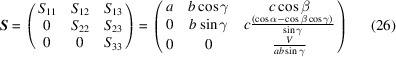

and its inverse is

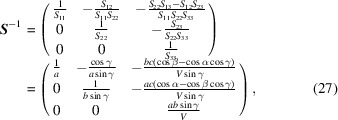

where the volume of the unit cell of crystal I is

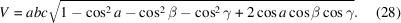

The modified O-lattice conversion matrix 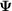 can be solved as

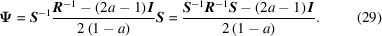

This indicates that finding a CSL for a non-cubic lattice requires modifying the rotation matrix by using its translation matrix. Note that the final result is expressed in the Bravais lattice basis, not in the reference basis. This is convenient and useful because a conversion between the reference coordination system and the Bravais lattice system is omitted in this formula.

## 4. Obtaining a CSL from the simplified O-lattice

Since the CSL is a superlattice of the simplified O-lattice, it is convenient to obtain the CSL by using a linear combination of the vectors of the O-lattice with a constraint that the volume should be *n* times that of the O-lattice unit cell. The integer number *n* is determined by equation (16). It is interesting to note that there is no quantitatively analytical solution to this step yet, although it looks quite simple. A few approaches to finding CSL vectors from a rotation matrix or an O-lattice have been reported and used (Grimmer *et al.*, 1974 ▸). However, they are based on trial-and error methods and careful choosing of intermediate parameters. Loquias & Zeiner (2010 ▸) gave a complete mathematical solution in terms of coincidence isometries of a shifted square lattice, but this was too difficult to be understood and accepted by materials scientists. This suggests possible future development of the CSL method.

## 5. Case studies

This section will demonstrate a few examples of CSL and NCSL calculations to validate the simplified O-lattice proposed above and to explore its applicability. The lattice-parameter ratios of the crystals in the following cases are denoted as LPRs. As is well known, CSLs can be found in a high-symmetry lattice, such as cubic and hexagonal, with specific LPRs. However, this is not always true for low-symmetry ones. It appears from the literature that the properties of the CSL (*e.g.* the Σ value) are not so closely related with the properties of the grain boundary for low-symmetry cases (Gertsman & Szpunar, 1999 ▸). Consequently, the concepts of a near coincidence site lattice (NCSL) and a constraint coincidence site lattice (CCSL) were introduced shortly after the difficulty of finding a CSL in a low-symmetry lattice was encountered.

The examples for low-symmetry lattices, such as ortho­rhombic and monoclinic lattices, are presented here to validate the simplified relationship between an NCSL rotation matrix and the corresponding O-lattice to the relaxed lattice. The other Bravias lattices are omitted here because a trigonal lattice can be treated as a hexagonal one, a tetragonal lattice is a special case of an orthorhombic one, and it is rarely easy to find a CSL or an NCSL for a triclinic lattice. However, the following is not an attempt to claim that a CSL can always be found in a low-symmetry lattice.

### 5.1. Case I: a Σ5 CSL of [100]/36.87° in a simple cubic structure (LPR *a*_0_)

For a simple cubic structure, a rotation of 36.87° around the [100] axis generates a Σ5 CSL. The generation parameter *a* = cos 36.87° = 4/5. The rotation matrix ***R*** is

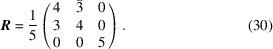

The conversion matrix 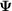 reads

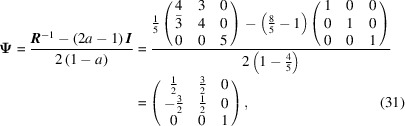




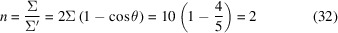

and

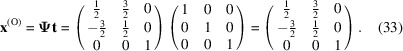

The CSL vectors are obtained by the vector operation on the condition that the unit cell of the modified O-lattice is multiplied by *n* = 2:

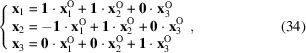




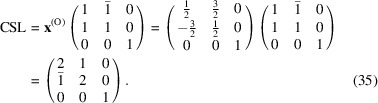




### 5.2. Case II: a Σ7 CSL of [111]/38.21° in a simple cubic structure (LPR *a*_0_)

For a simple cubic structure, a rotation of 38.21° around the [111] axis generates a Σ7 CSL. The parameter *a* = cos 38.21° = 11/14. The rotation matrix ***R*** is

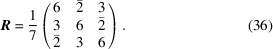

The conversion matrix 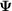 reads

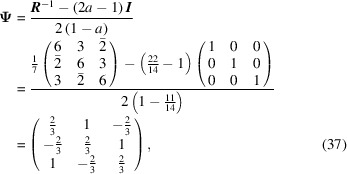




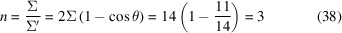

and

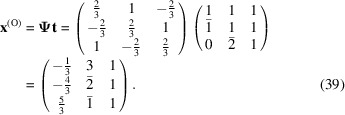

Finally, the CSL vectors are obtained in the same way and on the condition that the unit cell of the modified O-lattice is multiplied by *n* = 3:

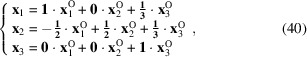




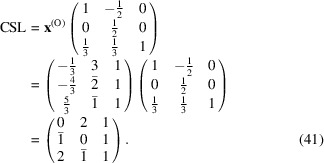




### 5.3. Case III: a Σ17 CSL of [100]/86.63° in a simple hexagonal structure [LPR (*c*_0_/*a*_0_)^2^ = 8/3]

For a simple hexagonal structure with LPR (*c*_0_/*a*_0_)^2^ = 8/3, a rotation of 86.63° around the [100] axis generates a Σ17 CSL where the parameter *a* = cos 86.63° = 1/17. The rotation matrix ***R*** is

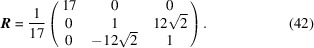

The transformation matrix ***S*** and its inverse for a hexagonal structure are

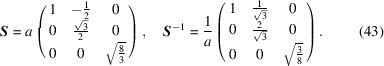

The conversion matrix 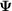 reads

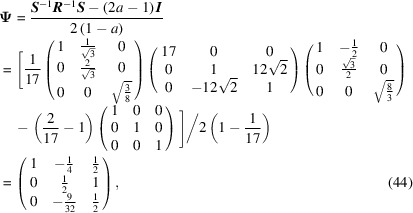




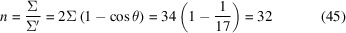

and

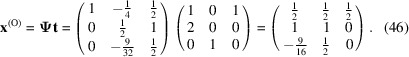

Finally, the CSL vectors are obtained by the operation on the condition that the unit cell of the modified O-lattice is multiplied by *n* = 32:

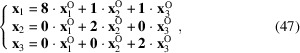




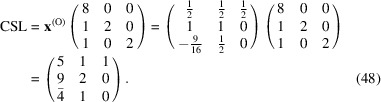




### 5.4. Case IV: a Σ3 CSL of [010]/48.3° in the orthorhombic LiFePO_4_ structure [LPR (*a*_0_/*b*_0_/*c*_0_)^2^ = 80/25/16]

It is not very common in the literature to investigate a CSL or an NCSL in orthorhombic crystals. What has been studied is the notable YBCO superconductor. However, this ortho­rhombic structure has a pseudo-tetragonal lattice with lattice parameters *a*_0_ = 3.82, *b*_0_ = 3.89 and *c*_0_ = 11.67 Å (for the nominal composition, *i.e.*, δ = 0, of YBa_2_Cu_3_O_7−δ_). As shown in Appendix A1, it is not a problem to verify the simplified O-lattice in this system.

Another example of this type of structure is lithium iron phosphate (or triphylite), LFP, LiFePO_4_. This is an environmentally friendly material (Padhi *et al.*, 1997 ▸) used as the cathode in lithium ion batteries (LIBs). This structure has an orthorhombic unit cell with space group *Pnma* and lattice parameters *a*_0_ = 10.329, *b*_0_ = 6.007 and *c*_0_ = 4.691 Å. The lattice parameters are far from pseudo-tetragonal. Kuriplach *et al.* (2019 ▸) composed a near-CSL Σ3 grain boundary with the (101) plane for this structure. The CSL is determined by translation vectors **a** − **c**, **b** and 3**c**:

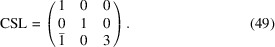

It was created by a rotation of 48.3° around the [010] axis. The parameter *a* = cos 48.3° = 2/3.

The corresponding rotation matrix ***R*** is 

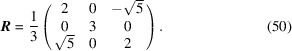

The lattice parameters are slightly relaxed by about 4% to obtain an NCSL. This relaxation is 

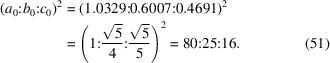

The transformation matrix ***S*** and its inverse for the ortho­rhombic LFP structure is

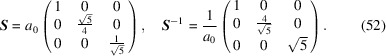

The conversion matrix 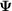 reads

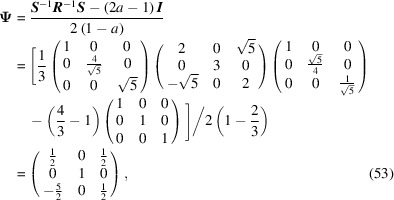




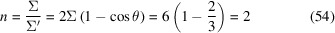

and

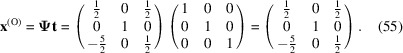

Finally, the CSL vectors are obtained by the operation on the condition that the unit cell of the modified O-lattice is multiplied by *n* = 2:

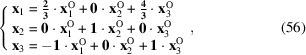




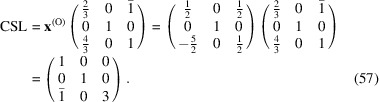

This CSL is identical to that reported by Kuriplach *et al.* (2019 ▸) It is obvious that the determinant of the multiplicity matrix is equal to 2:

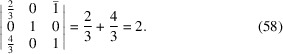




### 5.5. Case V: a Σ25 CSL of [010]/49.91° in the monoclinic sodium hydrogencarbonate (NaHCO_3_) structure (LPR *a*_0_/*b*_0_/*c*_0_ = 4/3/8)

A CSL or an NCSL in a monoclinic lattice is not common either. Monoclinic ZrO_2_ is often chosen as another model structure for a low-symmetry lattice NCSL calculation. However, this structure can be considered as pseudo-cubic, see Appendix A2.

To further validate the simplified O-lattice by using a strong case for a monoclinic structure, sodium hydrogen carbonate (NaHCO_3_) is chosen because it has a very large LPR. Its space group is *P*2_1_/*n* and its lattice parameters are *a*_0_ = 7.469, *b*_0_ = 9.684, *c*_0_ = 3.479 Å, β = 93.32°. A (101) twin has been observed in crystals of NaHCO_3_ (Aquilano *et al.*, 2015 ▸). The twin axis is [010]_m_ (where m indicates monoclinic cell) and the rotation angle is 49.91° for a Σ8 (101) twin. The (101) twin as an NCSL is investigated here. The analytical expression of the NCSL translation vectors was not described in the original work. The present author deduced it by using a stereographic projection tool (Liu & Liu, 2012 ▸). The matrix form of the CSL for the (101) twin is

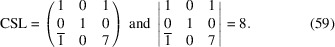

The parameter *a* = cos 49.91° = 0.6440 ≃ 5/8.

The transformation matrix ***S*** and its inverse for monoclinic NaHCO_3_ are

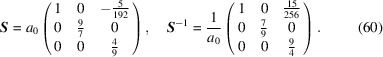

The rotation axis **u**_O_ (where O indicates orthorhombic basis) is converted from **u**_m_ used in the monoclinic basis:

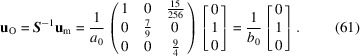

The rotation matrix ***R*** is then a 2D rotation around the *Z* axis of the orthorhombic basis:

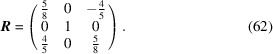

The conversion matrix 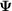 reads

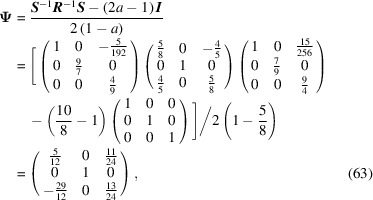




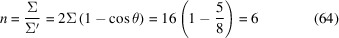

and

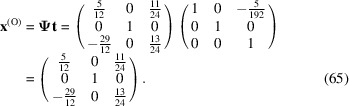

The value −15/192 in the translation vector **t** is due to a slight tilting of [001]_m_ away from the *Z* axis of the orthogonal basis by about β − π/2 = 3.32°. Obviously, its effect on the final modified O-lattice can be ignored because of its very small modulus. Finally, the CSL vectors are obtained by the operation on the condition that the unit cell of the modified O-lattice is multiplied by *n* = 6:

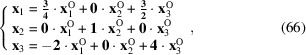




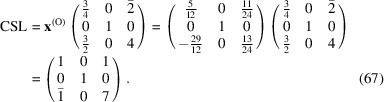

It is obvious that the determinant of the multiplicity matrix is equal to 6:

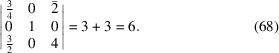




## 6. Discussion

The case studies above showed the successful application of the simplification to the modified O-lattice of crystal lattices with high symmetry (cubic and hexagonal structures) in terms of exact CSLs. However, when it is used in low-symmetry cases, for instance for orthorhombic and monoclinic structures, it immediately faces the same problem of finding superimposed points as when extending the classical CSL methods to low-symmetry structures. For these cases, it is geometrically meaningless to discuss CSLs where the LPR of the low-symmetry structure does not involve integers.

After adopting the NCSL and constraint CSL concepts, the simplification to the modified O-lattice is applicable to orthorhombic structures, as shown for the Σ3 CSL of [010]/48.3° in the orthorhombic LiFePO_4_ structure, even though this is not a CSL anymore. Note that the CSL here is a constraint CSL or an NCSL. It should be possible to extend this to a tetragonal structure, as a special case of an orthorhombic structure.

Surprisingly, when attempting to apply it to a monoclinic structure, such as ZrO_2_ or NaHCO_3_, is is still possible to find an NCSL. Careful relaxation of the lattice parameters of a low-symmetry lattice is necessary to optimize the LPRs and get a rational NCSL.

It is probably safe to claim that the simplified relationship between the modified O-lattice and the rotation matrix is valid for CSLs and NCSLs of Bravais lattices for which an ortho­rhombic sublattice can be found, which is usually possible for an arbitrary Bravais lattice.

## 7. Conclusions

By investigating the modified O-lattice for CSLs proposed by Karakostas, it was found that there is a strong and direct connection between the modified O-lattice and the rotation matrix for deducing a CSL. A simplified analytical method for generating the modified O-lattice for CSLs and NCSLs was then developed for any Bravais lattice using only the rotation matrix. The simplified O-lattice formula was used successfully to obtain a CSL from a rotation operation for a few examples of Bravais lattices covering high-symmetry cubic, medium-symmetry hexagonal and low-symmetry orthorhombic and monoclinic lattices. It should be convenient to apply in computer-aided crystallographic calculations or simulations of phase boundaries.

## Appendix A Two further examples for low-symmetry lattices

### A1. Case VI: a Σ59 CSL of [001]/89.03° in the simple orthorhombic YBCO structure [LPR (*a*_0_/*b*_0_)^2^ = 29/30]

YBa_2_Cu_3_O_7−δ_ is often chosen as a model structure for low-symmetry lattice CSL calculations. This structure has an orthorhombic unit cell with lattice parameters *a*_0_ = 3.82, *b*_0_ = 3.89 and *c*_0_ = 11.67 Å for the nominal composition, *i.e.*, for δ = 0. A (110) twin boundary was observed in this structure with a value of Σ = 64 when *a* ^2^:*b* ^2^ = 63:65 (Zhu & Suenaga, 1992 ▸) or 59 when *a* ^2^:*b* ^2^ = 29:30 (Gertsman, 1992 ▸). Here a rotation of 89.03° around the [001] axis is chosen to generate a Σ59 NCSL. The parameter *a* = cos 89.03° = 1/59.

The corresponding rotation matrix ***R*** is

The transformation matrix ***S*** and its inverse for a hexagonal structure is

The conversion matrix  reads

and

Finally, the CSL vectors are obtained by the operation on the condition that the unit cell of the modified O-lattice is multiplied by *n* = 116:

It is obvious that the determinant of the multiplicity matrix is equal to 116:

### A2. Case VII: a Σ25 CSL of [106]/73.7° in the monoclinic ZrO_2_ structure (LPR *a*_0_/*b*_0_/*c*_0_ = 1/1/1)

As mentioned in Section 5.5, monoclinic ZrO_2_ is often chosen as another model structure for low-symmetry lattice NCSL calculations because of its pseudo-cubic nature. This structure has lattice parameters *a*_0_ = 5.149, *b*_0_ = 5.213, *c*_0_ = 5.316 Å and β = 99.228°. To find an NCSL in this crystal, a lattice relaxation is applied to monoclinic ZrO_2_ so that *a* = *b* = *c* and cos β = −1/6, where β = 99.5941°. For this relaxed lattice, Gertsman *et al.* (1996 ▸) generated a long list of NCSLs. Here a rotation of 73.7° around the [106]_m_ axis (where m indicates monoclinic basis) is chosen to generate a Σ25 NCSL. The parameter *a* = cos 73.7° = 7/25.

The transformation matrix ***S*** and its inverse for monoclinic ZrO_2_ are

The rotation axis **u**_o_ in the orthorhombic basis is converted from the axis **u**_m_ in the monoclinic basis:

The rotation matrix ***R*** is then a 2D rotation around the *Z* axis of the orthorhombic basis:

The conversion matrix  reads

and

Finally, the CSL vectors are obtained by the operation on the condition that the unit cell of the modified O-lattice is multiplied by *n* = 48:

It is obvious that the determinant of the multiplicity matrix is equal to 48:
